# Analysis of the developing gut microbiota in young dairy calves—impact of colostrum microbiota and gut disturbances

**DOI:** 10.1007/s11250-020-02535-9

**Published:** 2020-12-28

**Authors:** Bui Phan Thu Hang, Ewa Wredle, Johan Dicksved

**Affiliations:** 1 Department of Animal Sciences and Veterinary Medicine, An Giang University, Long Xuyên, An Giang Province Vietnam; 2 Department of Animal Nutrition and Management, Swedish University of Agricultural Sciences, SE-75007 Uppsala, Sweden; 3 Vietnam National University-Ho Chi Minh, Ho Chi Minh City, Vietnam

**Keywords:** 16S Amplicon sequencing, Antimicrobials, Colostrum, Diarrhea, Feces

## Abstract

The aim of this study was to characterize the colostrum and fecal microbiota in calves and to investigate whether fecal microbiota composition was related to colostrum microbiota or factors associated with calf health. Colostrum samples were collected in buckets after hand milking of 76 calving cows from 38 smallholder dairy farms. Fecal samples were taken directly from the rectum of 76 calves at birth and at 14 days age. The bacterial community structure in colostrum and feces was analyzed by terminal restriction fragment length polymorphism for all samples, and the microbial composition was determined by 16S rRNA gene amplicon sequencing for a subset of the samples (8 colostrum, 40 fecal samples). There was a significant difference in fecal microbiota composition between day 0 and day 14 samples, but no associations between the microbiota and average daily gain, birth weight, or transfer of passive immunity. At 14 days of age, *Faecalibacterium* and *Butyricicoccus* were prevalent in higher relative abundances in the gut of healthy calves compared to calves with diarrhea that had been treated with antimicrobials. Colostrum showed great variation in composition of microbiota but no association to fecal microbiota. This study provides the first insights into the composition of colostrum and fecal microbiota of young dairy calves in southern Vietnam and can form the basis for future more detailed studies.

## Introduction

Colostrum is not only a source of IgG but can also be a source of bacteria for early establishment of the gut microbiota. It has been shown that feeding colostrum soon after birth enhances the colonization of total bacteria in the gastrointestinal tract of calves within the first 12 h compared with calves not given colostrum (Malmuthuge et al. 2015a). It has also been reported that fresh colostrum contains *Lactobacillus*, *Bifidobacterium*, *Escherichia* (Malmuthuge et al. 2015a), *Staphylococcus* spp., coliforms, and *Streptococcus* spp. (Lima et al. 2017). Thus, the bacterial composition of colostrum can be highly important for microbial colonization of the gut. In addition, colostrum contains certain oligosaccharides that are of importance of gut health, for example, by inhibiting the adherence of pathogens to the intestinal epithelial cells (Maldonado-Gomez et al. 2015) or by serving as growth substances for the establishing bacterial community (Yu et al. 2013; E Hernandez-Castellano et al. 2014; Fischer et al. 2018). Besides the effects of colostrum, exposure to antimicrobials early in life can also influence establishment of the gut microbiota (Oultram et al. 2015). As an example, administration of vancomycin to mice resulted in a reduction in *Firmicutes* and *Bacteroidetes* and a corresponding increase in the proportion of *Proteobacteria* (Cotter et al. 2012)*.*

Early development of the gut microbiota is suggested to be important for proper development of the immune system, and it has been shown in a study on pigs that early management is critical for shaping early gut microbial composition (Mulder et al. 2011). Calves with a higher abundance of *Faecalibacterium* spp., especially *Faecalibacterium prausnitzii*, at one week of age have been observed to show higher daily weight gain but also a lower diarrhea incidence during the first four weeks of life (Oikonomou et al. 2013).

Knowledge about early management and factors of importance for colonization of the gut microbiota in calves is limited. The purpose of this study was therefore to analyze the microbiota in colostrum and calf feces, and to investigate the relationship with calf health, diarrhea, and antimicrobial exposure.

## Materials and methods

### Farms and sampling

The study was conducted on small-scale dairy farms in Dong Nai province, southern Vietnam, from August to December 2014. The selection criteria were that the farm should have 5–10 dairy cows and have at least two calves born during the experimental period. In total, 76 dairy calves (34 female and 42 male, Holstein Friesian crossbreeds) from 38 farms were included in the study, and all these calves were offered 2–4 L bucket-fed colostrum within 4 h after birth. Calves were housed individually without bedding material. After the first day of life, the calves were fed milk from a bucket twice a day at milking time. The amount fed was 2–4 L per occasion for female calves and less than 2 L per occasion for male calves after 3 days age. The male calves were fed a mixture of concentrate and water from day 4. Body weight of calves was measured when calves were newborn and at 2 weeks of age using a digital scale (TANITA HD-380, Tanita Corporation, Japan). The daily weight gain of female and male calves was 0.75 kg and 0.54 kg per day, respectively. Ten percent of both female and male calves had an IgG serum level below 8.3%, indicating failure of passive immune transfer in these calves. The diarrhea was identified by daily observation where calves with typical symptoms as watery stools, then losing appetite, and slight fever. The quality of the colostrum was evaluated based on content of IgG, fat, and protein (for more information, see Hang et al. 2017). A digital Brix refractometer (PAL-1, Atago Co. Ltd., Tokyo 173-0001 Japan) calibrated with distilled water was used to estimate IgG level in colostrum and serum, following the procedure of Bielmann et al. (2010). In conjunction with sampling, each farm was visited, and the farmers were asked to complete a short questionnaire on calf management practices such as antimicrobial usage, time to separation from the dam, feeding, and calf health.

Colostrum was sampled within 4 h after calving, by hand milking into a bucket. The colostrum was mixed thoroughly, and then 15-mL aliquots were transferred to duplicate sets of sterile tubes, rapidly placed on ice, and transported to the laboratory. Fecal samples were collected directly from the rectum of calves within 4 h after birth (=D0) and at 14 days of age (=D14). The fecal samples were transferred to sterile tubes, immediately placed on ice packs, and transported to the laboratory. All colostrum and fecal samples were placed in a freezer at − 20 °C upon arrival at the laboratory and were stored frozen at − 20 °C until analysis.

### Sample analysis

#### DNA isolation

DNA was isolated from 0.2 g of feces sample using the QIAamp DNA Mini Kit (QIAGEN, GmbH, Hilden, Germany). The DNA isolation was performed according to the manufacturer’s protocol, with the exception that a bead beating step was added to the protocol to ensure full lysis of bacteria. The bead beating was carried out using 0.1 mm zirconium/silica beads for 2 × 60 s in a Mini Bead Beater 8 (BioSpec Products Inc., Bartlesville, Oklahoma, USA) at the “homogenize” setting. The isolated DNA was stored at − 20 °C until further analysis.

DNA was isolated from 0.5-mL aliquots of colostrum. Each colostrum aliquot was mixed with 0.5-mL nuclease-free water, and the tubes were centrifuged at 13,000×*g* for 5 min, after which the fat layer and the supernatant were carefully removed. The remaining pellet was used for DNA isolation using a Power Food Microbial DNA Kit (MO BIO Laboratories Inc., Carlsbad, USA) according to the manufacturer’s protocol. The DNA was stored at − 20 °C until further analysis.

No-template controls, i.e., DNA isolation reactions without any samples added, were processed in parallel to the DNA isolations to check for potential contamination of samples during the DNA isolation steps.

#### Terminal-restriction fragment length polymorphism

To assess the structure of the microbiota in colostrum and fecal samples, the molecular fingerprinting method terminal restriction fragment length polymorphism (T-RFLP) was used. Sample preparation for the T-FRLP analysis followed a procedure described previously (Dicksved et al. 2008). In brief, 16S rRNA gene amplicons were generated from the isolated DNA using the broad-range primers Bact-8F-FAM, end-labeled with a fluorescent dye (6-carboxyfluorescein), and Bact-926r. After confirmation of positive PCR reactions, the PCR products were digested with the HaeIII restriction enzyme (GE Healthcare, Uppsala, Sweden) at 37 °C for 2 h, and the digested fragments were separated on an ABI 3700 capillary sequencer (ABI, Applied Biosystems, Foster City, CA, USA). The size of the fluorescently labeled fragments was determined by comparison with the internal GS ROX-500 size standard (ABI). T-RFLP electropherograms were imaged using the Peak Scanner software (ABI), and relative peak area of each terminal restriction fragment (TRF) was determined by dividing the area of the peak of interest by the total area of peaks within the following threshold values: lower threshold at 50 bp and upper threshold at 500 bp.

#### Generation of 16S ribosomal RNA gene amplicon libraries

For a subset of the fecal samples, (20 samples collected from 14 days old calves that had been treated with antimicrobials and healthy 20 controls) and colostrum (selected based on differences in T-RFLP data, *n* = 8), the microbiota composition was analyzed in more detail by sequencing 16S rRNA gene amplicons with Illumina sequencing. The PCR amplicons were generated with barcoded universal primers (515F and 806R, amplifying the V4 region of the 16S gene). The PCR reactions were carried out using Phusion® High-Fidelity PCR chemistry (New England Biolabs, Ipswich, MA, USA). After confirmation of PCR products, samples were purified using Qiagen Gel extraction kit (Qiagen) and pooled in equimolar amounts. The amplicon libraries were processed with NEBNext Ultra DNA Library prep Kit, and the libraries were sequenced on Illumina HiSeq platform 2500 at Novogene (Beijing, China).

The raw reads generated were demultiplexed and assigned to different samples according to the respective barcode. The paired-end sequence reads were merged using FLASH (Version 1.2.7) (Magoč and Salzberg 2011). The merged reads, on average 417 bp and after truncating off the primer and barcode sequences the merged reads were then quality filtered according to the Split Libraries procedure in QIIME (Version 1.7.0) (Caporaso et al. 2010). The quality-filtered sequences were aligned to the Gold database (Release 20,110,519), and chimera sequences were detected and removed using the UCHIME Algorithm (Version 7.0.1001) (Edgar et al. 2011). UPARSE software (Version 7.0.1001) (Edgar 2013) was used to cluster the remaining sequences into operational taxonomic units (OTUs), using ≥  97% homology to be classified as an OTU. For each OTU, a representative sequence was selected for annotation of taxonomic information using the SSU rRNA database SILVA (http://www.arb-silva.se/).

#### Statistical analysis

Microbial community structures were compared with multivariate statistical models by conducting principal coordinate analysis (PCoA) based on Bray-Curtis distances. Analysis of similarity (ANOSIM) with Bray-Curtis distance matrices was used to test for differences between groups/exposures. Univariate statistical methods were used to test for differences in relative abundance between groups. The Mann-Whitney test was used when comparing the median between two groups, while the Kruskal-Wallis test was used when comparing more than two groups. Only microbial taxa (genus level) with an average relative abundance of > 1% were included in the univariate analysis. All statistical tests were performed using the statistical software Past (Hammer et al. 2001).

## Results

### Records of farm management and calf health

All farms had a similar management practice. All cows had been treated with antibiotics, ampicillin and cloxacillin*,* in the dry period. After birth, calves were immediately separated from their mothers and fed colostrum within 4 h of birth. Calf health and antimicrobial use is summarized in Table 1.

**Table 1:** Calf health and antimicrobial usage^*^ on smallholder dairy farms in southern Vietnam

Item	Female	Male	Total of calves
Diarrhea	2	12	14
Antimicrobial treatment due to diarrhea	2	12	14
Antimicrobial treatment of other reasons^**^	4	4	8

*The antimicrobials used on the farms: gentamicin, tylocine, tetracycline, ampicillin, oxytetracycline, or enrofloxacin

^**^Calves with symptoms of inflamed umbilicus, swollen joints, or fever

### Profiling of the microbiota in colostrum and feces

**Fig. 1:**
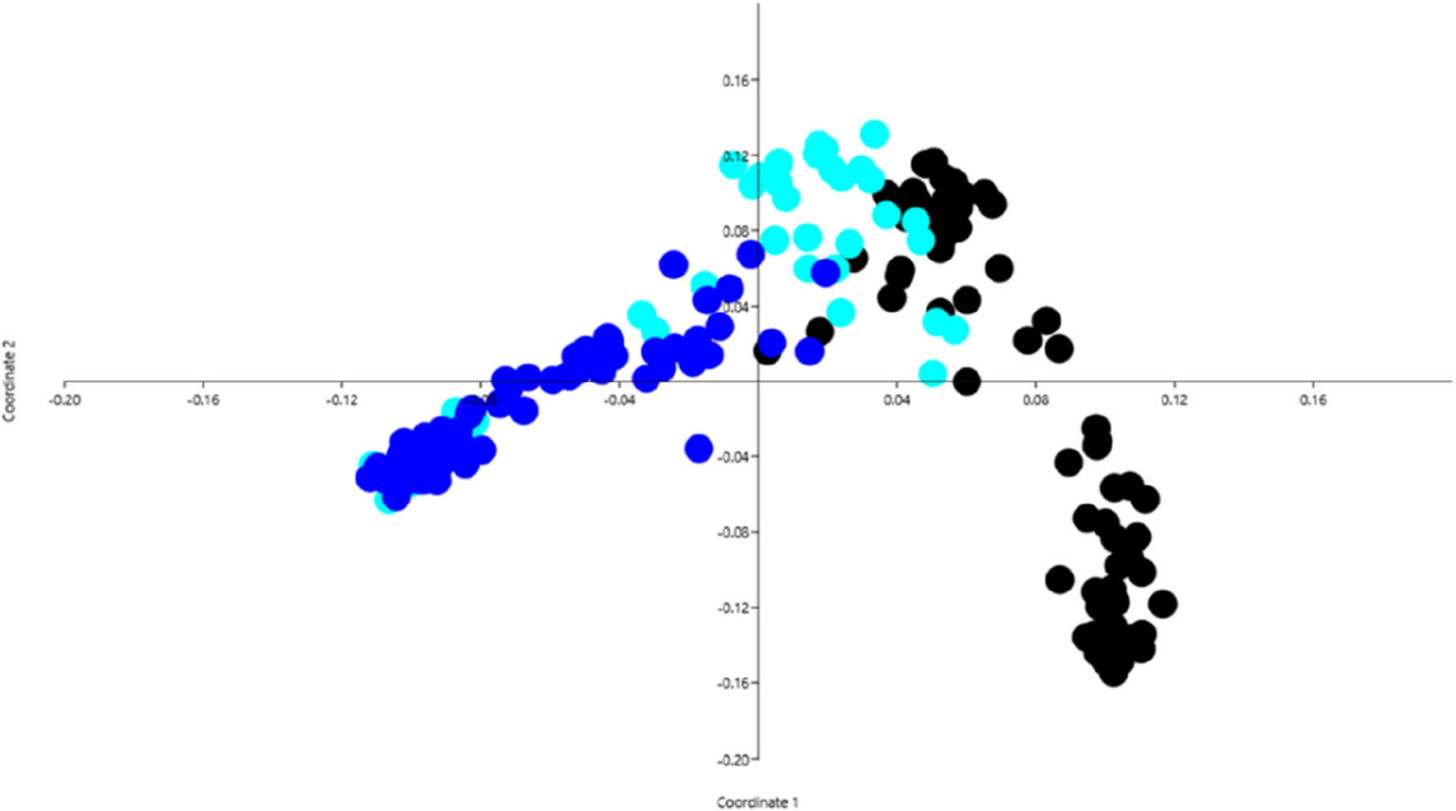
Principal coordinate analysis based on Bray-Curtis distances of data generated from the terminal restriction fragment length polymorphism (T-RFLP) analysis. Colors represent origin of samples: colostrum samples = black, feces samples at birth (D0) = light blue, and feces samples at 14 days of age (D14) = blue

T-RFLP was used to assess the structure of the microbiota in the collected colostrum and fecal samples. However, it was not possible to generate PCR amplicons from all samples. In particular, for the feces D0 samples, only 38 out of 76 samples generated PCR products, despite several attempts. For the colostrum and feces D14 samples, we had to exclude six and one samples, respectively, due to absence of successful PCR amplicons. The data generated from the remaining samples were assessed with multivariate statistical approaches. Principal coordinate analysis (PCoA), based on the Bray-Curtis distance metrics, was used to evaluate the data from the T-RFLP analysis and revealed separate clustering of colostrum samples and fecal samples (Fig. 1). Among the fecal samples, almost all D0 samples clustered together, but were separated from the D14 samples. The colostrum samples were more associated with the feces D0 samples, indicating higher similarity in composition of the microbiota. The colostrum samples were also divided into two clusters, indicating variation in the microbiota composition between different colostrum samples. ANOSIM was used to test for differences between the groups. This test confirmed that there were significant differences between the group categories feces-D0, feces-D14, and colostrum (*P* = 0.0001).

To test whether the microbiota profile was associated with other variables reported or measured for the calves, ANOSIM tests were used to search for associations between microbiota profile and gender, average daily gain (ADG), birth weight, transfer of passive immunity, and antimicrobial treatment. However, none of these variables, either for newborn calves (D0) or for 14-day-old calves, was linked to any clustering pattern of the fecal microbiota, and none was significant in the ANOSIM analysis.

### Composition of the microbiota in colostrum and feces

**Fig. 2:**
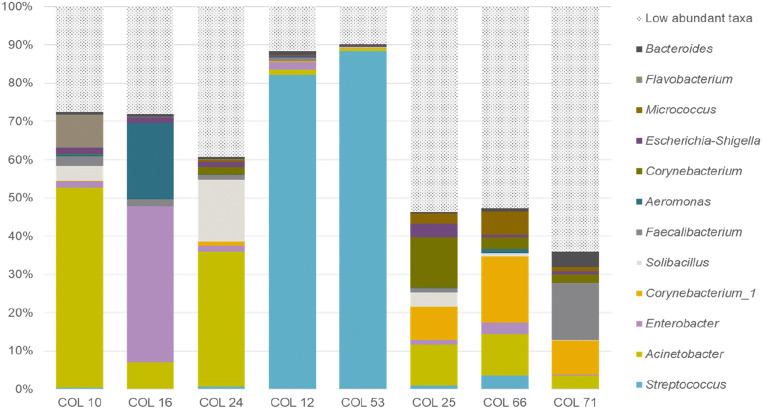
Proportions of the 12 most abundant taxa in colostrum samples, based on sequence data

**Fig. 3:**
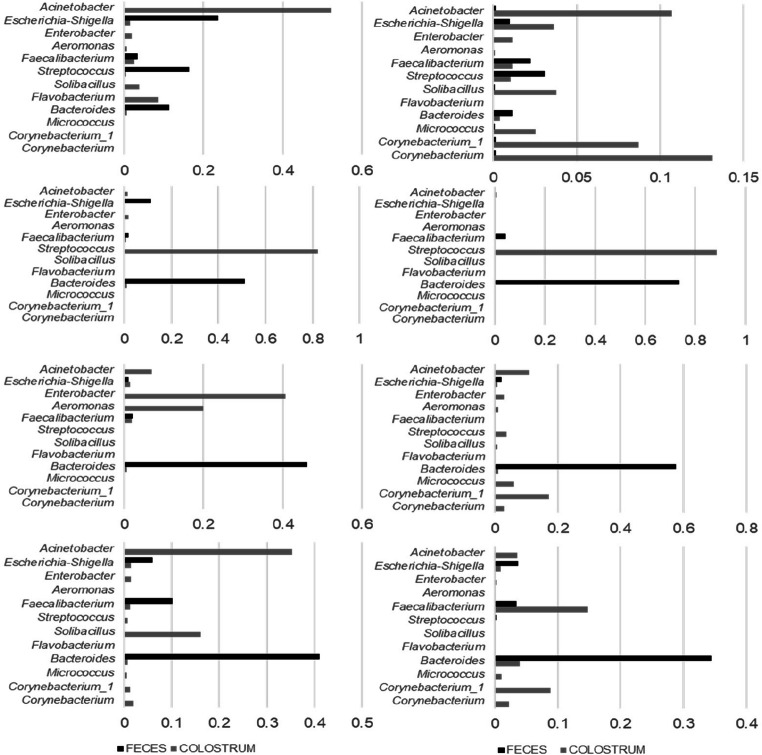
Relative abundance of predominant taxa between feces samples taken at 14 days of age and the corresponding colostrum sample fed to the calf. Each panel represents a sample pair, i.e., microbiota in colostrum and the corresponding feces sample

The T-RFLP analysis showed that the microbiota in colostrum differed in composition from that in feces (Fig. 1). To assess the microbial taxa present in colostrum and feces, the 16S rRNA gene was sequenced in eight colostrum samples and 40 fecal samples from the 14-day-old calves. The colostrum samples were selected based on differential clustering in the T-RFLP analysis. The fecal samples selected for sequence analysis were stratified into two groups; one group of healthy calves (*n* = 20) and one group where the calf had been treated with antimicrobials within the first two weeks of life (*n* = 20). The colostrum samples contained in average 38,154 (range 26,246–48,170) sequences per sample, whereas the fecal samples generated in average 40,251 (range 27,448–49,201) sequences per sample. Analysis of the microbial composition in the colostrum samples revealed a large variation in the composition of microbiota between different colostrum samples (Fig. 2). The colostrum microbiota was primarily dominated by facultative anaerobic bacteria such as *Streptococcus*, *Acinetobacter*, *Enterobacter*, and *Corynebacterium*. The dominant taxa found in colostrum samples were mostly also detected in the fecal samples from calves that had been fed the colostrum (Fig. 3). However, there were low associations in relative abundance of microbiota between the colostrum and fecal samples.

### Comparison of the microbiota in calves with and without gut disturbances

**Fig. 4:**
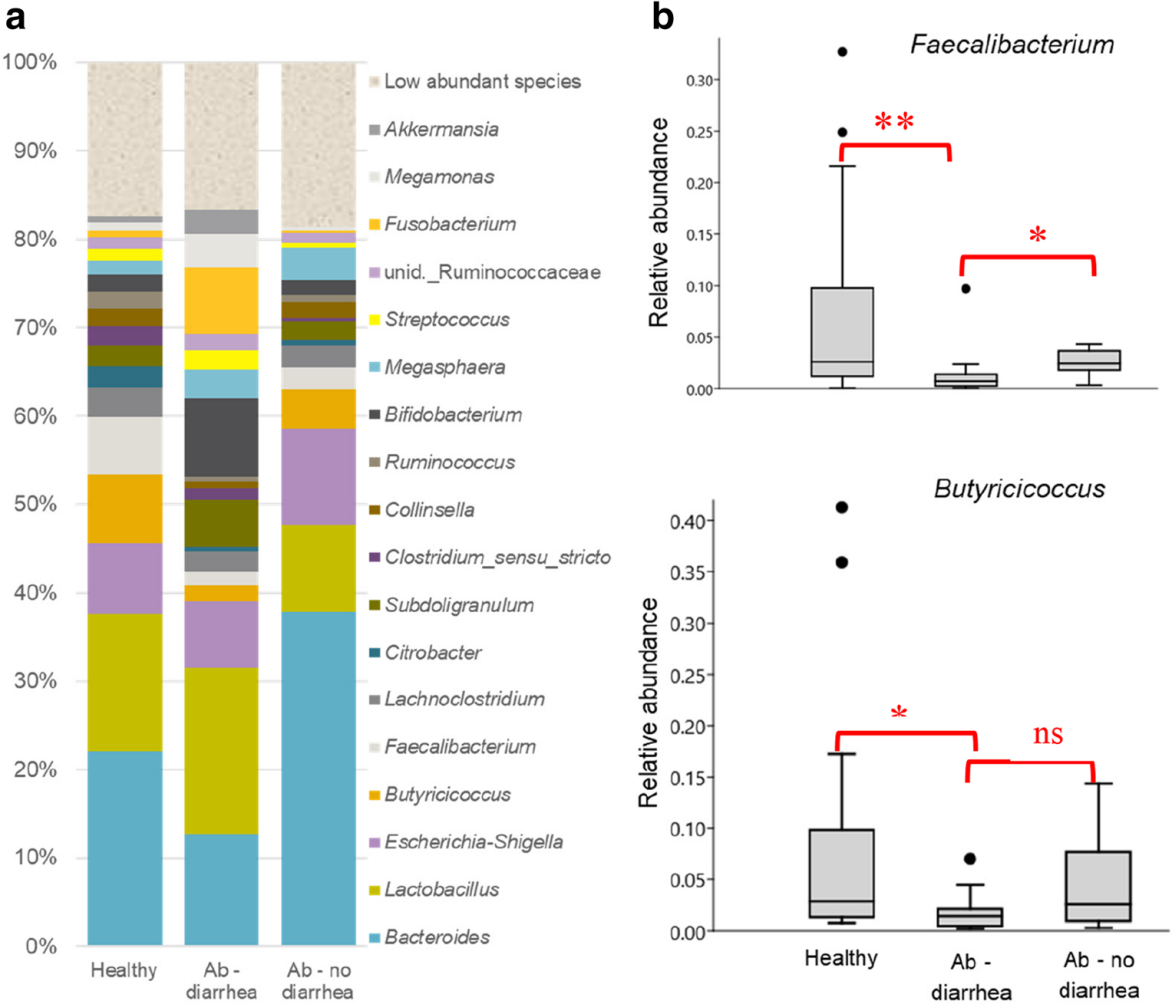
The microbiota in feces samples from 14-day-old calves, divided into three groups (healthy, Ab, diarrhea (antibiotic-treated with diarrhea), and Ab, no diarrhea (antibiotic-treated without diarrhea)). (**a**) shows distribution (average abundance for each group) of the main bacterial taxa found in samples. (**b**) shows the relative abundance of *Faecalibacterium* and *Butyricicoccus* in the three groups. * and ** indicate significant difference at *P* < 0.05 and *P* < 0.01, respectively

Sequence data was generated from 20 of the calves in this study that had been treated with antimicrobials within their first two weeks of life. Several tests were performed to determine whether the microbiota in these calves differed from that in healthy calves without any antimicrobial treatment. Comparing the 16S amplicon sequence data generated from these two groups did not reveal any significant differences in alpha diversity (based on Shannon’s diversity), while the ANOSIM analysis did not reveal any significant differences in composition. However, when calves treated with antimicrobials were further subdivided into whether they had diarrhea (*n* = 13, mainly the male calves) or not (*n* = 7), there were differences in composition between the groups (Fig. 4a), and the ANOSIM analysis revealed a significant difference between healthy controls and calves with diarrhea (*P* = 0.015). To identify the microbial taxa contributing to this difference, Kruskal-Wallis tests were applied and revealed a significant difference in relative abundance of *Butyricicoccus* (*P* = 0.014) and *Faecalibacterium* (*P* = 0.005) between healthy calves and those with diarrhea. Both *Faecalibacterium* and *Butyricicoccus* were present in lower relative abundance in the calves with diarrhea compared with healthy calves (Fig. 4b).

## Discussion

This study showed that the composition of the microbiota changes dramatically from birth and during the first two weeks of life. One source of bacteria and nutrients that could help to shape the initial composition of the gut microbiota is colostrum. The colostrum fed to the calf not only is nutrient-rich but also contains growth agents and antimicrobial factors (Kehoe et al. 2007). A recent study showed an association between the colostrum microbiota and fecal microbiota where *Streptococcaceae*, *Enterococcaceae*, and *Enterobacteriaceae* constituted up to 90% of the relative abundance in colostrum samples, whereas these groups were present in about 30% relative abundance in fecal samples at 7-days-old dairy calves (Liu et al. 2019). In southern Vietnam, dairy calves are removed from their mothers at birth but generally offered colostrum within 4 h of birth, using buckets as in the present study. In our data, the microbiota in colostrum had a community composition that clustered distinctly separately from that in the fecal samples, but with higher similarity between colostrum and D0 samples compared with D14 samples. High abundance of a species in colostrum did not mean that the taxon was present in high abundance in feces at D14. This was likely due to the development of the microbiota, which changes considerable during the first hours after birth (Malmuthuge et al. 2015b). Analysis of feces samples collected during the first 48 h after birth would have been very useful to study the effect of colostrum feeding on the intestinal microbiota during the first days of life. As this study was performed in several commercial farms, it was not possible to collect these fecal samples at this early stage of life. Our data showed that the microbiota in several of the colostrum samples had a more similar composition to D0 fecal samples than to D14 fecal samples, but it also showed large variation in microbiota composition between different colostrum samples. The microbiota detected in the colostrum samples was likely also impacted by other factors; for example, the colostrum samples in this study was collected directly from the buckets, which were not sterile and likely contributed with bacteria from the environment. Furthermore, intra-mammary antimicrobial therapy had been administered to all cows at drying off in our study, and this practice can affect the colostrum microbiota (Lima et al. 2017). The goal with the analysis of the colostrum samples was however not to analyze the colostrum microbiota per se but instead to investigate what microbes the calf ingests initially and that potentially could be the very early colonizers of the gastrointestinal tract.

The initial colonization of the gut microbiota was explored in order to identify factors that contributed to its composition and links between microbiota and health. However, 50% of the D0 samples were negative in the PCR, and it was not possible to get a PCR product in these samples, despite several attempts. This was probably due to too low numbers of bacteria. Although the issue has been debated and the data are somewhat conflicting, the gut is considered to be sterile in utero (Malmuthuge et al. 2015b). Due to the early sampling of the rectum in calves in this study, it can be assumed that no bacteria had yet passed through the gastrointestinal tract and reached the rectum at the time of D0 sampling. However, none of the collected samples were sterile, since we had previously isolated *E. coli* from the D0 samples on collection (Hang et al. 2019). Yet, we cannot rule out that the detected bacteria in the D0 samples derived from the sample collection procedure. It is certainly plausible that bacteria located in the surrounding skin around the rectum were captured in the sampling procedure.

Around 60% of dairy calf deaths before weaning are reported to be caused by infections resulting in diarrhea (Uetake 2013). Oikonomou et al. (2013) reported that a high prevalence of *Faecalibacterium* spp. early in life is associated with less diarrhea in newborn calves. In the present study, we found that healthy calves had a significantly higher relative abundance of *Faecalibacterium* and *Butyricicoccus* in feces compared with calves that suffered from diarrhea. Diarrhea was observed in more cases for male calves (12/14 diarrhea calves) most likely because they were fed concentrate mixed with water from 4 days age during the study period (Hang et al. 2017). Calves with diarrhea had also been treated with antimicrobials, which could have an impact on the microbiota composition (Oultram et al. 2015). However, we did not detect any difference in the animals treated with antimicrobials without diarrhea, despite similar exposure to antimicrobials. *Faecalibacterium* and *Butyricicoccus* are part of the normal microbiota in various species, and *Faecalibacterium* in particular has been linked to health. High prevalence of *Faecalibacterium* in the first week of life has also been associated with decreased incidence of diarrhea and increased weight gain in calves. Moreover, *F. prausnitzii* has been shown to be important for maintaining intestinal homeostasis (Oikonomou et al. 2013). The findings presented here provide further information regarding the potentially beneficial effect of *Faecalibacterium* in newborn calves and its association with health.

*Butyricicoccus* was also significantly more abundant in feces of healthy calves in the present study, and this was also shown by Tomassini (2015). Alipour et al. (2018) noted that *Butyricicoccus* and *Faecalibacterium* species are commonly positively correlated in the gut. One shared feature of *Faecalibacterium* and *Butyricicoccus* is the ability to produce butyrate in the gut. Butyrate is not only a nutrient source for the gut colonocytes, but it is also thought to be beneficial for the immunological maturation of the gut mucosa (Furusawa et al. 2013).

Our study demonstrated a significant age-dependent shift in the gut microbiota of young dairy calves. Colostrum is a source of bacteria and nutrients for the developing gut. At 14 days of age, there were differences in abundances of *Faecalibacterium* and *Butyricicoccus* between calves with gut disturbances and healthy calves.
